# Fabrication and electrical properties of MoS_2_ nanodisc-based back-gated field effect transistors

**DOI:** 10.1186/1556-276X-9-100

**Published:** 2014-02-28

**Authors:** Weixia Gu, Jiaoyan Shen, Xiying Ma

**Affiliations:** 1 School of Mathematics and Physics, Suzhou University of Science and Technology, 1# Kerui Road, Suzhou, Jiangsu 215009, China

**Keywords:** Molybdenum disulfide, CVD, Field effect transistors, Mobility

## Abstract

Two-dimensional (2D) molybdenum disulfide (MoS_2_) is an attractive alternative semiconductor material for next-generation low-power nanoelectronic applications, due to its special structure and large bandgap. Here, we report the fabrication of large-area MoS_2_ nanodiscs and their incorporation into back-gated field effect transistors (FETs) whose electrical properties we characterize. The MoS_2_ nanodiscs, fabricated via chemical vapor deposition (CVD), are homogeneous and continuous, and their thickness of around 5 nm is equal to a few layers of MoS_2_. In addition, we find that the MoS_2_ nanodisc-based back-gated field effect transistors with nickel electrodes achieve very high performance. The transistors exhibit an on/off current ratio of up to 1.9 × 10^5^, and a maximum transconductance of up to 27 μS (5.4 μS/μm). Moreover, their mobility is as high as 368 cm^2^/Vs. Furthermore, the transistors have good output characteristics and can be easily modulated by the back gate. The electrical properties of the MoS_2_ nanodisc transistors are better than or comparable to those values extracted from single and multilayer MoS_2_ FETs.

## Background

The structure of molybdenum disulfide (MoS_2_), a layered transition metal dichalcogenide (TMD), comprises S-Mo-S in a hexagonal close-packed arrangement. Covalent bonds exist between the atoms in each layer, while the layers interact via weak van der Waals forces. Similar to extracting graphene from graphite [1], bulk MoS_2_ is easily split into single-layer (SL) or few-layer (FL) MoS_2_ sheets. Compared with graphene, single and multilayer MoS_2_ have a larger bandgap [2-6]. The presence of a large bandgap makes MoS_2_ more attractive than gapless graphene for logic circuits and amplifier devices. Single and multilayer MoS_2_ field effect transistors (FETs) have been prepared with on/off current ratio exceeding 10^8^ at room temperature, effective mobility as high as 700 cm^2^/Vs and steep subthreshold swing (74 mV/decade) [7-13]. MoS_2_ also shows great promise for optoelectronics [14,15] and energy harvesting [16,17] and other nanoelectronic applications.

MoS_2_ sheets are most commonly fabricated by micromechanical exfoliation (Scotch-tape peeling) [18,19]. Lithium-based intercalation [20,21], liquid-phase exfoliation [22], and other methods [23-25] have also been used to synthesize single-layer and few-layer MoS_2_. However, the yield and reproducibility of micromechanical exfoliation are poor, and the complexity of the other methods presents disadvantages to their use. Chemical vapor deposition (CVD) is a simple and scalable method for the synthesis of transition metal dichalcogenide thin films having large area. Liu et al. and Zhan et al. have successfully synthesized large-area MoS_2_ films via CVD [26,27].

Much research has been done on single and multilayer MoS_2_ FETs where the MoS_2_ layer is fabricated by micromechanical exfoliation then transferred to Si substrates. However, few studies have addressed the electrical properties of back-gated MoS_2_ field effect transistors with Ni as contact electrodes. This study is the first to report back-gated FETs based on MoS_2_ nanodiscs synthesized directly using CVD. The MoS_2_ nanodiscs fabricated via CVD are large and uniform. We herein report upon their surface morphologies, structures, carrier concentration, and mobility, as well as the output characteristics and transfer characteristics of FETs based on these obtained MoS_2_ nanodiscs, with Ni as contact electrodes.

## Methods

**Figure 1:**
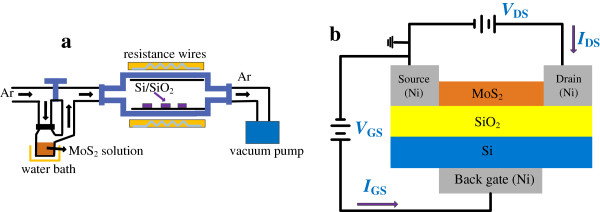
**Schematic view of experimental setup and MoS**_**2 **_**nanodisc-based back-gated FET. (a)** Schematic view of the experimental setup of CVD. **(b)** MoS_2_ FET with 50-nm-thick Ni as contact electrodes together with electrical connections. The channel is the MoS_2_ nanodiscs, and 280-nm SiO_2_ serves as gate dielectric. The length and width of the channel are 1.5 and 5 μm, respectively.

MoS_2_ nanodiscs were deposited via CVD on n-type silicon (111) substrates covered with a 280-nm SiO_2_ layer. Figure 1a illustrates the CVD experimental setup, which is composed of five parts: a temperature control heating device, a vacuum system, an intake system, a gas meter, and a water bath. The Si substrates were placed in the center of a horizontal quartz tube furnace, after being ultrasonically cleaned with a sequence of ethanol and deionized water and dried with N_2_. A MoS_2_ solution was formed by adding 1-g analytical grade MoS_2_ micro powder to 200 mL of diluted sulfuric acid with stirring for 5 min at room temperature. The solution was then moved in a beaker flask that was placed in a water bath with a constant temperature of 70°C to improve the solubility of the powder. Before deposition, the furnace was evacuated to 10^−2^ Pa and heated to 300°C for 10 min to remove moisture. To deposit the MoS_2_ film, Ar gas with a volume ratio of 10 to 30 sccm was flowed into the MoS_2_ solution, carrying MoS_2_ molecules into the furnace's reactive chamber, which was kept at a constant temperature of 550°C and a working pressure of 50 Pa for 10 min to obtain uniform growth. The nanodiscs were formed by the adsorption and deposition of MoS_2_ molecules onto the SiO_2_/Si substrates. To improve the quality of the discs, and their ability to form electrical contacts, the samples were further annealed at 850°C for 30 min in Ar. Finally, the furnace was slowly cooled back down to room temperature and the samples were removed. Some of the MoS_2_ discs were set aside as representative samples for characterization of surface morphologies and structures, and the others were used to fabricate MoS_2_ back-gated FETs.

Figure 1b is a schematic of a MoS_2_ back-gated FET. The source and drain electrodes were formed by lithographic patterning, and Ni electrodes were sputtered onto them using magnetron sputtering technology. The MoS_2_ nanodiscs serve as the channel, whose length and width are 1.5 and 5 μm, respectively. The back gate of the FET was completed by sputtering a 50-nm-thick Ni layer on the back of the Si substrate.

The surface morphology and crystalline structure of the MoS_2_ discs were analyzed by atomic force microscopy (AFM) and X-ray diffraction (XRD), respectively. The electrical properties of the samples were measured using a Hall Effect Measurement System (HMS-3000, Ecopia, Anyang, South Korea) at room temperature. The electrical properties of the MoS_2_ nanodisc-based FETs, configured as shown in Figure 1b, were measured using a Keithley 4200 semiconductor characterization system (Cleveland, OH, USA).

## Results and discussion

**Figure 2:**
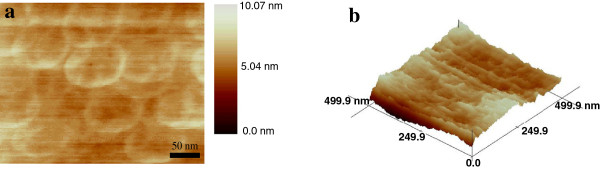
**AFM image and three-dimensional distribution of the MoS**_**2 **_**film. (a)** An AFM image of the MoS_2_ nanodisc film deposited on the SiO_2_/Si substrate. **(b)** Three-dimensional distribution of the MoS_2_ nanodiscs.

Figure 2a shows the AFM topographic image of the MoS_2_ discs deposited on the Si substrates. The MoS_2_ nanodiscs are round and flat, with a diameter of 100 nm and a thickness of around 5 nm, which is equal to the thickness of a few MoS_2_ layers. The uniform color of the MoS_2_ nanodiscs in the AFM image, as well as the line profile corresponding to a cross section of the sample, indicating that the nanodiscs all have approximately equal thickness. Figure 2b shows a three-dimensional image of the MoS_2_ nanodisc film, which further confirms the high quality of the MoS_2_ nanodisc film.

**Figure 3:**
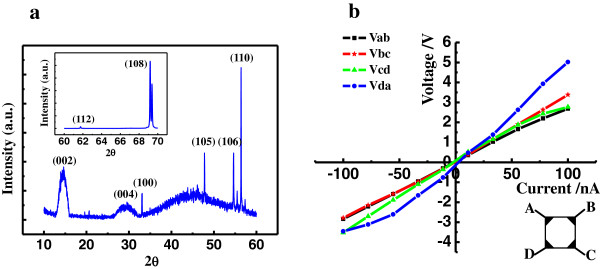
**Properties of the MoS**_**2 **_**nanodiscs. (a)** XRD pattern of the obtained MoS_2_ nanodiscs for the diffraction angle in the range of 10° ~ 60°. Inset: the diffraction spectrum of MoS_2_ nanodiscs for the diffraction angle in the range of 60° ~ 70°. **(b)** The surface current-voltage curves of the MoS_2_ nanodiscs. Inset: the layout of four measured points on the MoS_2_ disc film.

Figure 3a shows XRD patterns of the obtained MoS_2_ nanodiscs. Because the intensities of the diffraction peaks differed too widely to be presented in a single plot, the larger plot shows the diffraction peaks in the range of 10° to 60°, while the small insert shows the diffraction peaks that appear between 60° and 70°. Over the whole range of diffraction angles, the MoS_2_ nanodiscs exhibit eight diffraction peaks, located at 14.7°, 29.5°, 33.1°, 47.8°, 54.6°, 56.4°, 61.7°, and 69.2°. They are assigned, respectively, to the diffraction planes (002), (004), (100), (105), (106), (110), (112), and (108) of MoS_2_ according to data from the JPDS. The presence of these peaks demonstrates that the obtained MoS_2_ nanodiscs exhibit a variety of crystal structures. Moreover, the obtained diffraction peaks are rather sharp, which shows that the MoS_2_ nanodiscs are crystalline over a large area. The peak corresponding to the (108) crystal face is much more intense than the other peaks, indicating that the discs have a strong tendency to adopt the (108) crystal orientation during their growth.

The surface current-voltage (*I*-*V*) properties, surface carrier concentration and mobility of the obtained MoS_2_ nanodiscs are very sensitive to the quality of the film. Figure 3b shows the surface *I*-*V* properties of the MoS_2_ nanodisc film. The inset shows the layout of the four measurement points on the MoS_2_ nanodisc film. The *I*-*V* curves measured between any two points show a perfect linear dependence, which indicates that the deposited MoS_2_ nanodiscs have good conductivity. The measured carrier concentration of the MoS_2_ discs is about 3.412 × 10^6^ cm^−2^, and their electron mobility is as high as 6.42 × 10^2^ cm^2^/Vs. This mobility value is higher than previously reported values (2 to 3 × 10^2^ cm^2^/Vs) for single and multilayer MoS_2_[19,28]. This significant increase of room-temperature mobility value in our MoS_2_ may result from the MoS_2_ nanodisc structure. The mobility of SL MoS_2_ is generally smaller than bulk MoS_2_ because of the larger phonon scattering [29]. However, FL MoS_2_ exhibits fewer dangling bonds and defect states than does SL MoS_2_, significantly decreasing the phonon scattering. The lattice scattering in the two-dimensional (2D) nanodiscs should be even lower, due to their surface roughness and boundaries. The above findings clearly demonstrate that the MoS_2_ nanodiscs fabricated via CVD have uniform morphologies, structures, and electrical properties.

**Figure 4:**
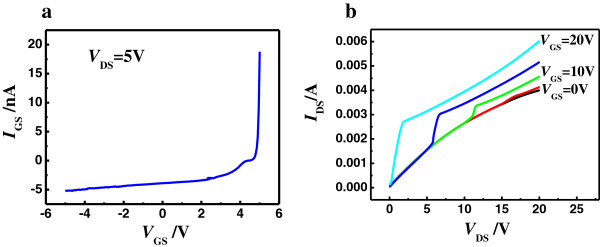
**The current–voltage behavior of back-gated MoS**_**2 **_**transistor. (a)** Gate current *I*_GS_ versus gate voltage *V*_GS_ behavior of back-gated MoS_2_ transistor at room temperature for the drain voltage *V*_DS_ value of 5 V. **(b)** Output characteristics of back-gated MoS_2_ transistors at room temperature for *V*_GS_ values of 0, 5, 10, 15, and 20 V.

The electrical properties of the MoS_2_ nanodisc-based back-gated FETs, with Ni as the source, drain, and back gate contacts were next investigated at room temperature. Figure 4a shows the relationship between the gate current (*I*_GS_) and the gate voltage (*V*_GS_) of the transistor at a drain voltage (*V*_DS_) of 5 V. The current through the device increases exponentially with the applied positive voltage, and tends to be almost zero under the revised voltage, showing that the MoS_2_ transistor is a good rectifier.

Figure 4b displays the output characteristics (drain current *I*_DS_ versus drain voltage *V*_DS_) of back-gated MoS_2_ transistors at room temperature for *V*_GS_ = 0, 5, 10, 15, and 20 V. For small *V*_GS_, the current *I*_DS_ shows an exponential dependence on *V*_DS_ at low *V*_DS_ values, which results from the presence of a sizable Schottky barrier at the Ni-MoS_2_ interface [12]. Then, for larger values of *V*_GS_, the relation between *I*_DS_ and *V*_DS_ becomes linear as *V*_DS_ increases, which is consistent with the previously reported findings [12]. The barrier height at larger *V*_GS_ is lower that has been previously demonstrated in greater detail [12,30,31]. Thus, the channel can give rise to thermally assisted tunneling, which is responsible for the linear relationship between *I*_DS_ and *V*_DS_. Finally, when *V*_DS_ increases above a certain value, the current *I*_DS_ becomes saturated, achieving the output properties of a traditional FET.

**Figure 5:**
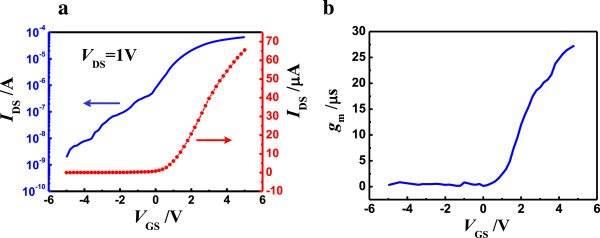
**Transfer characteristics of back-gated MoS**_**2 **_**transistor (a) and device transconductance versus gate voltage (b). (a)** Transfer characteristics of MoS_2_ transistor at room temperature for the *V*_DS_ value of 1 V on logarithmic (left axis) and linear scales (right axis). **(b)** Device transconductance *g*_m_ (defined as *g*_m_ = d*I*_DS_/d*V*_GS_) versus gate voltage *V*_GS_ at *V*_DS_ = 1 V.

Figure 5a shows the transfer characteristics (*I*_DS_/*V*_GS_) of the back-gated MoS_2_ transistor at room temperature for *V*_DS_ = 1 V. It is clear that the gate leakage of the FET is negligible and the on/off current ratio can be up to 1.9 × 10^5^, larger than that in the WSe_2_-based FETs at low temperature [32], which demonstrates that the MoS_2_ transistor can be easily modulated by the back gate. Moreover, the Fermi level of Ni is close to the conduction band edge of MoS_2_, consistent with earlier reports [7,12], which makes MoS_2_ transistors exhibit mostly n-type behavior. Figure 5b shows the variation of the device transconductance *g*_m_ (*g*_m_ = d*I*_DS_/d*V*_GS_) with *V*_GS_ at *V*_DS_ = 1 V. The extracted maximum *g*_m_ is about 27μS (5.4 μS/μm) within the entire range of *V*_GS_, better than previously reported values [7,12]. The field effect mobility μ also can be obtained based on the conventional dependence of μ = *g*_m_ [*L*/(*W · C*_OX_ *· V*_DS_)] at *V*_DS_ = 1 V, where *g*_m_ is the maximum value of *g*_m_, and *L* and *W* are the length and width of the channel, and *C*_OX_ = 1.1 × 10^−4^ F/m^2^ is the gate capacitance per unit area [33]. *C*_OX_ is equal to *ϵ*_OX_/*d*_OX_, where *ϵ*_OX_ is the dielectric constant and *d*_OX_ is the thickness of the gate dielectric. Using this relationship, the field effect mobility μ is as high as 368 cm^2^/Vs, comparable to that of single and multilayer MoS_2_ FETs [7,10,12,26,34]. Note that the field effect mobility is lower than the electron mobility of the MoS_2_ nanodiscs, which is likely due to the presence of scattering and defect states.

## Conclusions

Using CVD, we have fabricated uniform MoS_2_ nanodiscs, organized into thin films with large area and having good electrical properties. The nanodiscs were incorporated into high-performance back-gated field effect transistors with Ni as contact electrodes. The transistors have good output characteristics and exhibit typical n-type behavior, with a maximum transconductance of approximately 27 μS (5.4 μS/μm), an on/off current ratio of up to 1.9 × 10^5^ and a mobility as high as 368 cm^2^/Vs, comparable to that of FETs based on single and multilayer MoS_2_. These promising values along with the very good electrical characteristics, MoS_2_ transistors will be the attractive candidates for future low-power applications.

## Competing interests

The authors declare that they have no competing interests.

## Authors’ contributions

WG participated in the fabrication of MoS_2_ nanodiscs on the substrate, measured the electrical properties of the transistor, and wrote the manuscript. JS fabricated the drain, source, and gate of the transistor and participated in the analysis of the results of the transistor. XM designed the structure of the transistor and analyzed the results. All authors read and approved the final manuscript.

## Authors’ information

WG is a graduate student major in fabrication of new semiconductor nanometer materials. JS is a lecturer and PhD-degree holder specializing in semiconductor devices. XM is a professor and PhD-degree holder specializing in semiconductor materials and devices, especially expert in nanoscaled optical-electronic materials and optoelectronic devices.
